# Starting strong: development and biomechanics of the seedling–host interaction in European mistletoe (*Viscum album*)

**DOI:** 10.1093/jxb/eraf129

**Published:** 2025-05-07

**Authors:** Luiza Teixeira-Costa, Lukas Wiese, Thomas Speck, Max D Mylo

**Affiliations:** Work Group on Wildness, Biodiversity and Ecosystems Under Change, Vrije Universiteit Brussel, Brussel, Belgium; Plant Biomechanics Group, Botanic Garden Freiburg, University of Freiburg, Freiburg, Germany; Department of Microsystems Engineering—IMTEK, University of Freiburg, Freiburg, Germany; Plant Biomechanics Group, Botanic Garden Freiburg, University of Freiburg, Freiburg, Germany; Cluster of Excellence livMatS, FIT—Freiburg Center for Interactive Materials and Bioinspired Technologies, University of Freiburg, Freiburg, Germany; Plant Biomechanics Group, Botanic Garden Freiburg, University of Freiburg, Freiburg, Germany; Cluster of Excellence livMatS, FIT—Freiburg Center for Interactive Materials and Bioinspired Technologies, University of Freiburg, Freiburg, Germany; Department of Microsystems Engineering—IMTEK, University of Freiburg, Freiburg, Germany; University of Cambridge, UK

**Keywords:** Haustorium, microtomography, mistletoe, parasitic plant, plant biomechanics, Santalaceae, seed attachment, seedling development, tensile test

## Abstract

Attachment to a substrate is fundamental for plant growth and development. This is especially true for species that live either partially or fully off the ground, such as mistletoes, which have developed unique adaptations to anchor themselves securely to host trees from which they draw water and some nutrients. While the mechanical properties of attachment during the adult stages in many plant species have been described, the mechanical principles of the initial developmental stages are rarely investigated. Here, we focus on the parasitic European mistletoe (*Viscum album* L.) and its attachment to a host plant at the seedling stage. Using a combination of germination experiments, microtomography, histological analysis, and biomechanical tests, this work investigates the role of the three key attachment structures involved in this process: the seed coat, hypocotyl, and holdfast. The viscin layer, a sticky coating on the seed, provides initial adhesion before the growing hypocotyl expands towards the host surface, where it flattens and forms a holdfast that strengthens adhesion and aids tissue penetration. Tensile tests revealed that these three attachment structures withstand similar forces in the early stages, considerably higher than the weight of the seedling. Within a few months, the endophytic system interlocked with the host bark, forming a robust connection that not only transports water but also increased the mechanical strength of the structure. This work highlights the fundamental mechanisms of the initial mistletoe–host interaction, which forms the basis of their decades-long relationship.

## Teaser

Mistletoe seedlings form a strong attachment to the host already at the seedling stage by combining an interlocking mechanism with the hypocotyl, forming a bridge-like structure that strengthens the parasite–host connection.

## Graphical Abstract

**Graphical Abstract:**
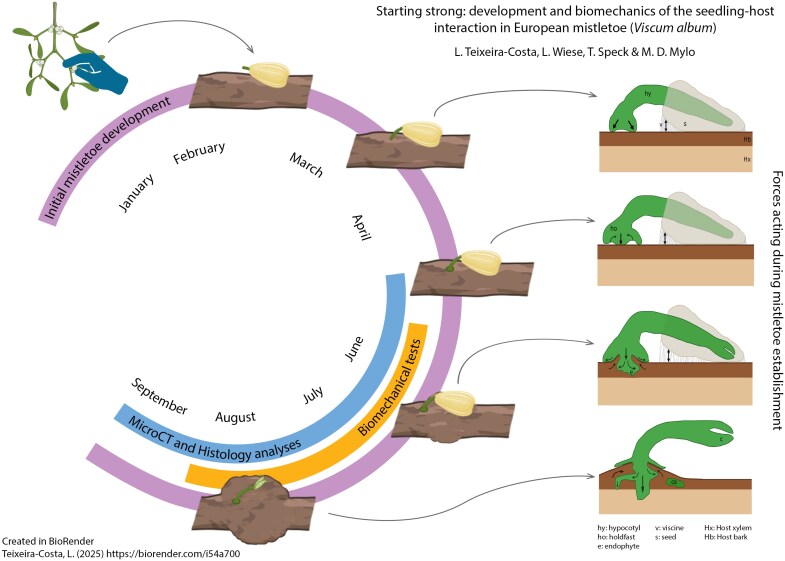

## Introduction

One of the main functions of plant root systems is to anchor the plant to a substrate (Gorb, 2008). This is as crucial for fully terrestrial plants rooted in soil, as for those growing with partial or no direct contact with soil, including climbing plants, epiphytes, parasitic vines, and mistletoes. To fulfil a partially or fully ‘aerial’ lifestyle, these plants display a wide range of morphological adaptations (Rowe *et al*., 2004). Plants that germinate on the ground and later climb up a substrate often develop structures such as hooks, tendrils, and adhesive pads (Melzer *et al.*, 2010; Seidelmann *et al.*, 2012; Rowe and Speck, 2015; Klimm *et al.*, 2022, 2024; Lehnebach *et al*., 2022). In several species, specialized secretory cells produce adhesive substances that cover the substrate and provide initial binding for the aerial plant (Groot *et al.*, 2003; Tay *et al.*, 2022). Moreover, surface interlocking mechanisms are frequently observed in epiphytes that grow on top of a substrate to secure a strong attachment (Tay *et al.*, 2023). Indeed, these attachment mechanisms are vital for high-canopy epiphytes, as their chances of survival are remarkably lower than those of their low-canopy counterparts if substrate connection is lost (Spicer and Ortega, 2023).

Aerial parasitic plants depend even more on a strong attachment, as is the case for parasitic vines *Cuscuta* spp. (Convolvulaceae) and the mistletoe *Viscum album* (Viscaceae). Due to their parasitic lifestyle, these plants must not only attach, but also penetrate, and quickly establish contact with the vascular tissue of the host plant, from which they derive all or part of their nutrients (Kuijt, 1969). These crucial functions are accomplished by an organ termed the haustorium, which develops as a mosaic with both root and stem characteristics (Teixeira-Costa, 2021). In both *Cuscuta* spp. and *V. album*, initial attachment to the host surface is promoted by pectin-rich substances (Sallé, 1983; Vaughn, 2002). Following attachment, initial haustorium formation is triggered by a combination of light quality and availability, as well as physical contact with the substrate (Lamont, 1983; Tada *et al.*, 1996). Finally, as indicated by transcriptome analyses, the secretion of a variety of cell wall hydrolase enzymes facilitates penetration of host tissues and the establishment of vascular connections between parasite and host (Ko *et al.*, 2014; Olsen and Krause, 2017).

Despite similarities in their initial haustorium attachment and development processes, parasitic vines and mistletoes have fundamentally different life histories (Teixeira-Costa and Davis, 2021). *Cuscuta* species germinate in the soil and later coil around a host plant, developing haustoria on the concave side of the coiling region (Furuhashi *et al.*, 2011). On the other hand, *V. album* germinates directly upon host branches, which poses a greater risk of seed predation, desiccation, and dislodgement (Lamont, 1983). Moreover, the coiling habit of *Cuscuta* and its ability to trigger the expression of a binding protein on the host surface provide this parasite with additional mechanical support during host attachment and penetration (Tada *et al.*, 1996; Albert *et al.*, 2006). In *V. album* and other mistletoes, initial attachment is facilitated by the viscin layer coating the seed inside the fruit, which acts as a glue that binds the seed to the host branch (Aukema, 2003; Horbelt *et al*., 2019). Several other functions have been associated with this viscin coating, including protection from desiccation and host recognition (Gedalovich and Kuijt, 1987; Lichter and Berry, 1991). Nevertheless, its effect on haustorium penetration and contact establishment remains unknown in many aspects.

Here, we focus on the biomechanical aspects of these precarious, but crucial early developmental stages in the life cycle of the mistletoe *V. album*. We analyse the mechanical contributions of the seed coat and its viscin layer, the hypocotyl, and the holdfast in these initial stages and hypothesize that as development progresses, each of these structures in turn assumes a crucial role for securing attachment to the host branch. To better understand this mechanical interaction at the tissue level, we also provide a detailed morphological analysis of seedling development from germination to the formation of vascular contact between mistletoe and host.

## Materials and methods

### Plant material

To document seed germination and to analyse seedling development, a total of 72 ripe berries were collected from two female mistletoes (*V. album* subsp. *album*) that were growing on a flowering dogwood tree (*Cornus florida*, Cornaceae) in the Botanic Garden Freiburg, Germany. Berries were collected between January and February 2023. The first set of berries (36 in total) was collected from one mistletoe plant and were placed on the host branches without prior treatment. The other half of the collected berries were collected from a second plant and were soaked in hydrogen peroxide (H_2_O_2_) overnight, a method reported to improve germination rates in some mistletoe species (Lamont, 1983). Two host species were chosen: a quince tree (*Cydonia oblonga*, Rosaceae) located in the public outdoor area of the Botanic Garden, protected by a small fence, and two young apple trees (*Malus domestica*, Rosaceae) growing outdoors in the non-public part of the Botanic Garden. The status of each seed was monitored for 33 weeks. During early stages, external morphological characteristics of the berries and seedlings were monitored, classified, and photographed (Lumix DMC-FZ1000, Panasonic Corporation, Kadoma, Japan) on a weekly basis; as seedlings developed, they were monitored fortnightly, then monthly. Temperature data throughout the germination and growth stages were sourced from the Freiburg Faculty of Environment and Natural Resources, with the measuring station located at the Phenological Garden Freiburg, ~2 km straight-line distance from the Botanic Garden. Hourly temperature values were averaged over a week and the maximum value for the week was determined.

To maintain an adequate number of intact samples for seedling developmental analysis, two additional batches of mistletoe seedlings were used for the mechanical tests of the young mistletoe–host interaction. These samples were harvested from a maple tree (*Acer saccharum*, Sapindaceae) also growing in the Botanic Garden Freiburg. Sampling of the first batch took place between June and August 2023, shortly after the seedlings had penetrated the host branch. The second batch was sampled in November 2023, when hypertrophy of the host branch was evident in all samples. Care was taken to ensure that only healthy looking mistletoe seedlings were collected.

It is noteworthy that *V. album* subsp. *album* has one of the widest host ranges among parasitic plants, including >400 species (Barney *et al.*, 1998). In this scenario, some species are likely to be better hosts than others, as has been documented for other parasitic plants (Kuijt, 1969). Nevertheless, recent analyses have shown that key functional traits in *V. album* subsp. *album* plants do not vary according to host identity, suggesting that the parasite can adjust its physiology to survive on different hosts and in different site conditions (Wang *et al.*, 2023). Therefore, we consider all host species selected for this study to be equally capable of fulfilling the mistletoe nutritional requirements.

### Microtomography

For microtomography scanning, four seedlings growing on the apple trees were collected. In all samples, the holdfast was already permanently adhered to the host branch and their seeds were still attached to the host shoot. However, the samples differed in the degree of hypertrophy of the host branch and the general shape of the holdfast, which indicated that they covered the initial stage of host penetration. Following harvest, the samples were fixed in an FAA solution (63% ethanol, 27% distilled water, 5% acetic acid, 5% formalin) and then scanned. Alternatively, some samples were placed in a Lugol’s solution (1 g of potassium oxide, 0.5 g of iodine, 50 ml of distilled water) for ~1 month and stored in a desiccator under vacuum at room temperature prior to scanning (Teixeira-Costa, 2022). All samples were rinsed under running water and dried under a fume hood for ~1 h prior to scanning.

X-ray microtomographic (microCT) analyses were conducted using a Bruker scanner (Skyscan 1772) and the associated software (Skyscan, both Bruker Corporation, Billerica, MA, USA). Samples immersed in FAA were scanned without a filter, while samples immersed in Lugol’s solution were scanned with a 0.5 mm aluminium filter. All scans were acquired at a resolution of 7 µm, a voltage of 70 kV, and a current of 142 µA as 360° scans with a step size of 0.3°. Nrecon software (version 1.6.10.1, Micro Photonics Inc., Allentown, PA, USA) was used to reconstruct the scans with 2-pixel smoothing, beam hardening correction, and ring artefact correction. CTVox software (version 3.3.0, Bruker Corporation) was used to capture images of the resulting 3D geometry.

Tissue segmentation and video creation were conducted on scans of a 21-week-old and a 24-week-old sample using Avizo software (version 2022.2, Thermo Fisher Scientific, Berlin, Germany). The 21-week-old sample was differentiated into four segments: host branch, mistletoe seed, mistletoe hypocotyl, and mistletoe penetration tissue (endophyte). For the more developed 24-week-old sample, a distinction was also made between host wood that had grown regularly and wood that had grown as a result of hypertrophy. The ‘Brush’ and ‘Lasso’ tools were implemented to assign the individual pixel areas to the tissues in the cross-sections of the host branch and, where possible and under manual control, the ‘Interpolate’ function was used over several cross-sections.

### Histology

To better understand the composition of the plant tissues formed during different developmental stages, five samples were selected for anatomical analyses. Like the samples for microCT analysis, these samples represented different stages of the initial phase of host penetration, which were roughly determined by the degree of hypertrophy of the host branch. Each sample was gently boiled in a 5% glycerine solution (in distilled water) to help soften the host wood tissue. Next, samples were thoroughly washed in distilled water and placed in a solution of polyethylene glycol 6000. Sample blocks were sectioned at 20 µm using a semi-automatic rotary microtome (CUT 5062, SLEE medical GmbH, Nieder-Olm, Germany) with a conventional C-type knife. Histological sections were stained with safranin and astra blue, and mounted as permanent slides (Ruzin, 1999). Analysis and image acquisition were carried out with a Keyence VHX5000 microscope (Mechelen, Belgium) and a BX61 microscope, both equipped with a digital camera (DP71) using the cellP software (v2.8, all Olympus, Tokyo, Japan).

### Biomechanics

**Fig. 1.**
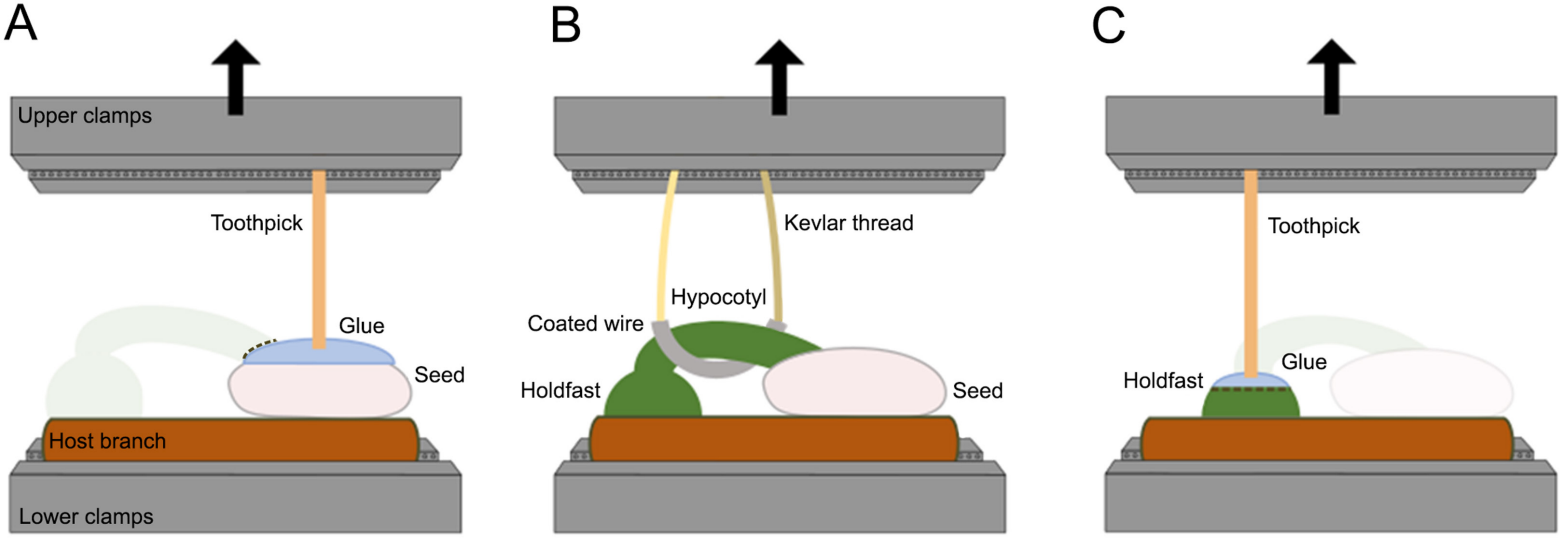
Sketch of the experimental setup for the biomechanical analyses, divided according to the mistletoe organ studied. In all experiments, the host branch was gripped with the lower clamps of the testing machine. (A) For seed experiments, the mistletoe holdfast was removed (dashed line) and the seed was glued to a toothpick through which the force was applied. (B) For the hypocotyl experiments, a coated wire was threaded through the hypocotyl loop, connected to Kevlar threads and thus fixed to the upper clamps of the testing machine. (C) For the holdfast experiments, the mistletoe hypocotyl was removed (dashed line) and the holdfast was glued to a toothpick, through which the force was applied.

The biomechanical tests were divided into three groups according to which organ of the mistletoe seedling (seed, holdfast, or hypocotyl) was investigated. For tests on seed and holdfast structures, samples were cut prior to testing to isolate the respective organ, whereas tests on the hypocotyl were carried out on unprepared samples (Fig. 1). All mechanical tests were performed under room conditions. To minimize the influence of humidity, samples were not harvested on rainy days and, in the case of heavy rainfall, samples were not collected for several days. Irrespective of the organ analysed, a uniaxial, displacement-controlled tensile load was applied at 2 mm min^–1^ until failure using a universal testing machine (Inspekt min.; Hegewald & Peschke Meß- und Prüftechnik GmbH, Nossen, Germany) equipped with a 100 N load cell. For the seed and holdfast tests, the attachment diameter of the respective organ was measured in two perpendicular directions before the experiment using a digital calliper (Mitutoyo Absolute Digimatic, measuring accuracy: ±0.03 mm, Kawasaki, Japan), and the contact area *A*_contact_ was calculated as an elliptical approximation.

The host branch was fixed in the lower clamps of the testing machine for all tests on all organs. For seed tests, toothpicks (with the tapered end removed) were glued (UHU Booster LED-Light, UHU GmbH & Co KG, Bühl, Germany) to the top of the mistletoe seed (Fig. 1A). This glue was used because it was very easy to apply and provided the best adhesion to the freshly cut surfaces. To ensure that only seed attachment was measured, the hypocotyl was cut off from the sample using a razor blade. The glue was dried by exposure to blue LED light and the toothpick was firmly gripped in the upper clamps of the testing machine, ensuring that the force applied was perpendicular to the contact surface of the seed and host branch. Soft paraffin was applied to the area around the seed beforehand to prevent overflowing gluing liquid from sticking to the host branch. For hypocotyl experiments, a plastic-coated wire was threaded through the arch of the hypocotyl and attached to a Kevlar thread, which was secured to the upper clamps that applied the tensile force (Fig. 1B). For analysing the holdfast structure, a method similar to seed testing was used. The holdfast was cut off at the transition to the hypocotyl using a razor blade and the resulting cut surface was then allowed to dry for a few minutes before being glued to the toothpick (Fig. 1C). A total of 36 successful experiments were carried out on the seed (all from the first batch), 17 experiments on the hypocotyl (13 in the first batch and 4 in the second batch), and 24 experiments on the holdfast (13 in the first batch and 1 in the second batch).

Samples that failed at the adhesive bond (glue) between the mistletoe organ and the toothpick, or that slipped noticeably at the clamps, were excluded from further analysis. For successful tests, the location and type of failure were documented, and the maximum force *F*_max_ [N] was extracted from the resulting force–displacement data. Statistical analysis was performed using Origin software (OriginPro 2023, OriginLab Corporation, Northampton, MA, USA). Due to small sample sizes in some groups (*n*≥3), non-parametric data were assumed for two-sided statistical comparisons between the different types of failure location within the groups of tested organs, and the Wilcoxon/Mann–Whitney test was performed. To statistically compare the maximum forces between the different organs tested, the data from each group were tested for normal distribution (Shapiro–Wilk test) and homogeneity of variance (Levene test). As the assumptions for parametric tests were not met (non-normal distribution in all groups), groups were compared using the Kruskal–Wallis test with Dunn post-hoc test. The alpha level for statistical significance was set to α=0.05 for all analyses.

We performed Pearson’s correlation analysis between the maximum forces and the cross-sectional areas of the seeds and the holdfasts to test for linear correlation between the variables. The resulting Pearson ρ values were classified according to Kampowski *et al.* (2017).

## Results

### Seedling development

**Fig. 2.**
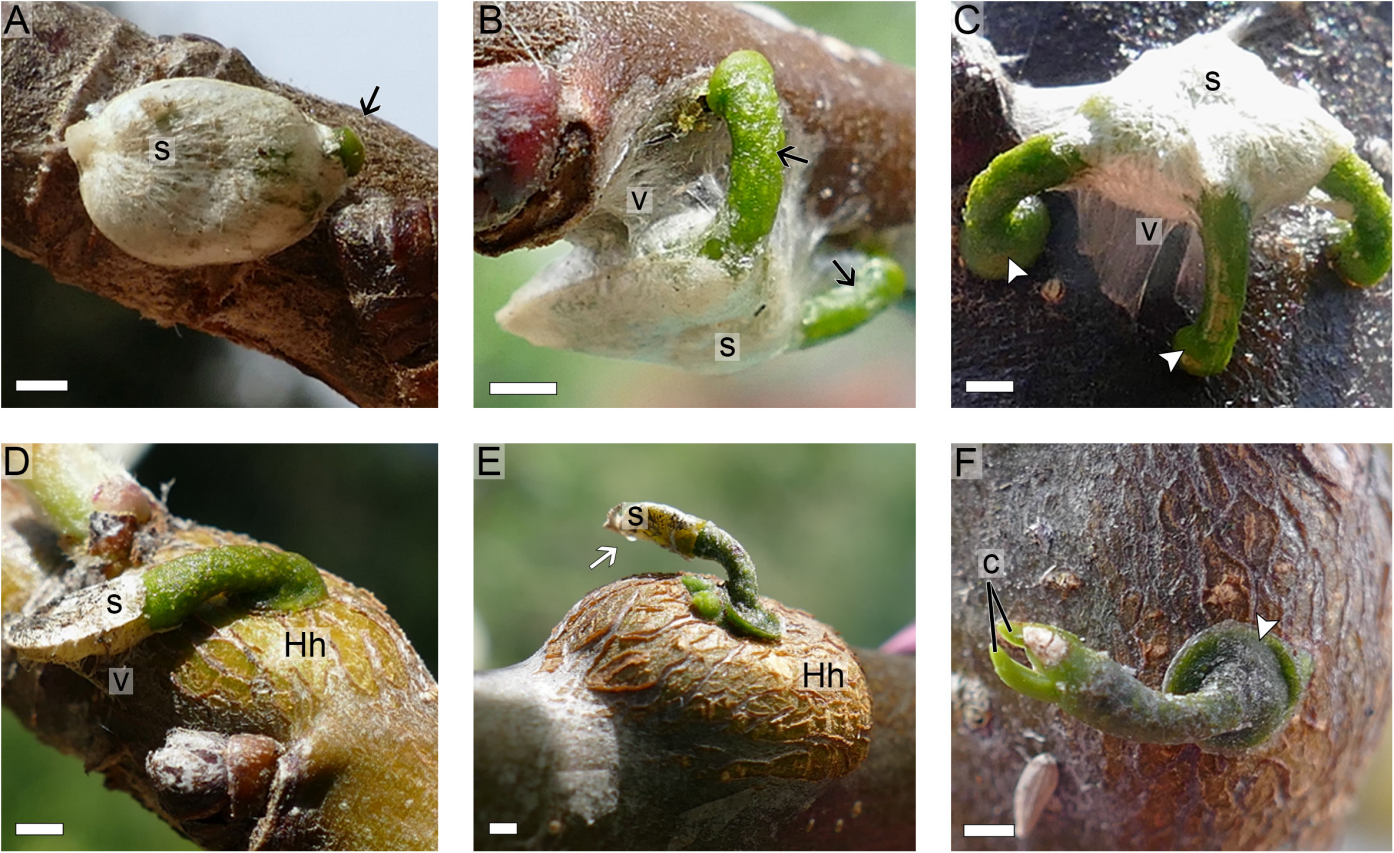
Seedling development. (A) Hypocotyl (arrow) protruding from the seed (s) marking successful germination. (B) Initial contact between hypocotyl (black arrows) and host branch. Notice abundant viscin (v). (C) Establishment of holdfast (arrowheads). Abundant viscin (v) still attaching the seed (s). Notice the dome-shaped holdfast (arrowheads). (D) Initial hypertrophy on host branch (Hh). Some viscin (v) is still present and the seed (s) remains attached to the host branch. (E) Seed (s) detachment from the host branch (white arrow). Notice increased host hypertrophy (Hh). (F) Cotyledon (c) release from seed coat. Notice flattening of the holdfast (arrowhead). All scale bars=1 mm.

Germination, determined by hypocotyl protrusion from the seed and marking the first developmental stage (Fig. 2A), was observed for 61 out of the 72 seeds sown (84.7%). The remaining seeds either detached from the host branch or became desiccated. H_2_O_2_ treatment of the berries increased the germination rate from 69.4% (untreated) to 97.2% (H_2_O_2_ treated) and reduced the initial duration until the hypocotyl grew from an average of 7 weeks (untreated) to 4 weeks (H_2_O_2_ treated). A detailed overview of the H_2_O_2_ treatment and other influencing factors such as the placement of the seeds above or below the host branch and the number of embryos per seed can be found in Supporting Fig. S1 and text available at the Zenodo data repository (doi.org/10.5281/zenodo.14179266). Successfully germinated seedlings remained attached to the host branch at this first stage exclusively by the viscin, which coated the seed inside the fruit. During this early development, the only noticeable morphological change was hypocotyl elongation, which culminated in the contact with the host surface, marking the beginning of the second developmental stage (Fig. 2B). Next, the tip of the hypocotyl spread against the host surface forming a dome-shaped structure known as the holdfast, which was about twice the diameter of the hypocotyl (Fig. 2C). This marked the beginning of a third developmental stage in which the holdfast provided additional contact between parasite and host. At this point, additional sample losses occurred due to death of the hypocotyl and thus the entire seedling.

After the holdfast was established, we observed a marked hypertrophy on most host branches in the region adjacent to the point of seedling attachment (Fig. 2D). This hypertrophy was caused by the development of mistletoe tissue inside the host bark (i.e. endophyte) following penetration. At this fourth developmental stage, few viscin filaments were visible, and those that were present were mostly desiccated. Moreover, the holdfast became visibly more flattened, thus increasing the contact area with the host surface and partially losing its initial dome shape. Seedlings growing on the quince tree did not reach this stage and were therefore presumed to have failed at penetrating host tissues. Among the seedlings growing on the two apple trees, further morphological changes were observed, including seed detachment from the host branch (Fig. 2E) and cotyledon release from the seed capsule (Fig. 2F). Further details on the germination process, including the duration of each developmental step (Supporting Fig. S1) and sampling dates (Supporting Fig. S2) can be found at Zenodo.

### Morphology

**Fig. 3.**
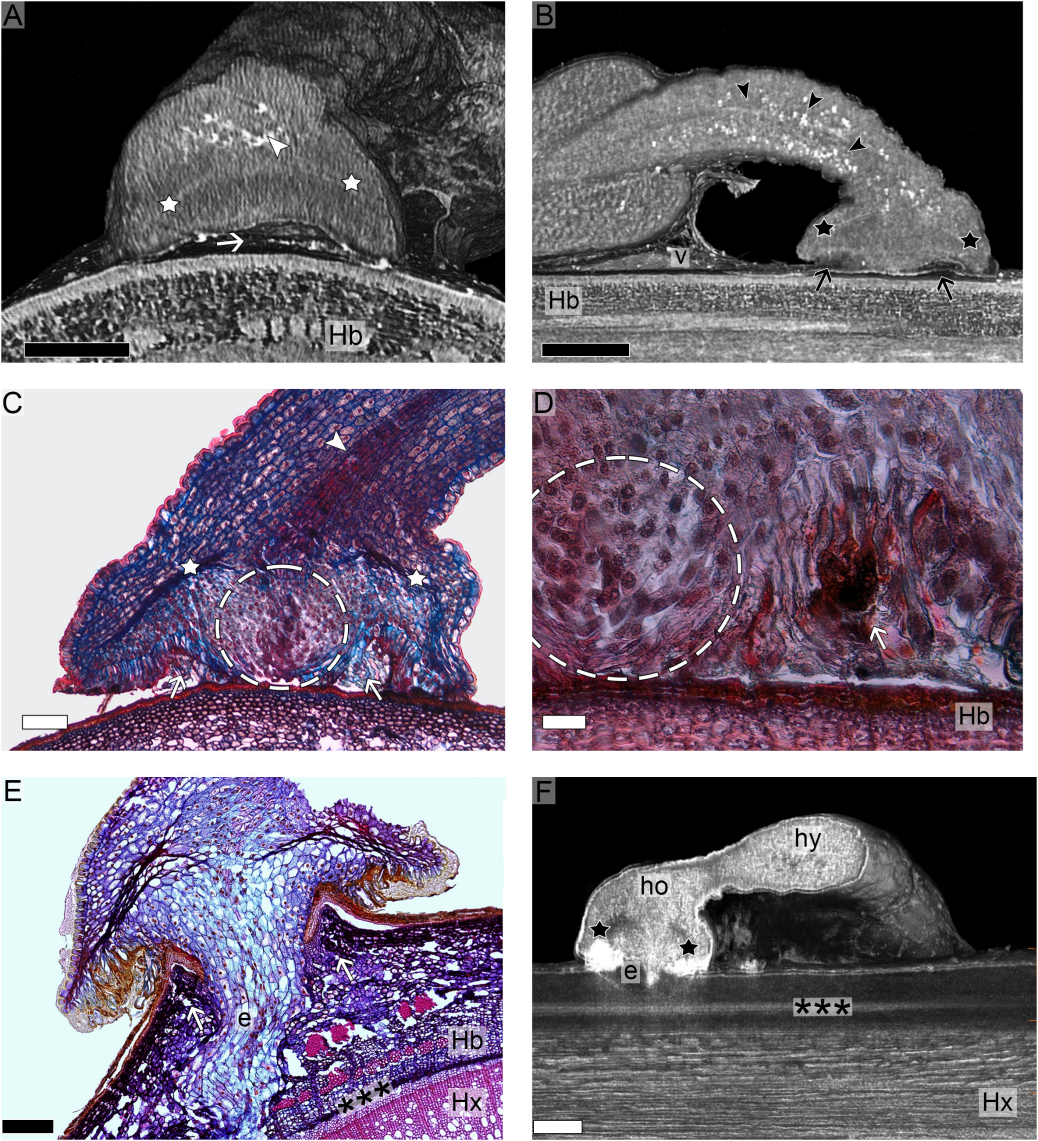
Morphology of early seedling development. (A) MicroCT scan showing a cavity (arrow) formed by the dome-shaped holdfast against the host bark (Hb). Notice high-density spots (arrowhead) visible above a high-density row of cells (stars). (B) MicroCT scan showing high-density spots (arrowheads) across the hypocotyl and holdfast visible above a high-density row of cells (stars). Penetration peg in the central area of the holdfast in contact with the host bark (Hb) forming a cavity ring (arrows). Notice viscin (v) remnants. (C) Longitudinal section through the seedling showing vessels (arrowhead) in the hypocotyl and in the upper part of the holdfast above two rows of crushed cells (stars), which flank an area of high meristematic activity (dashed circle). (D) Detail from Fig. 1C showing the holdfast with dark-stained cell wall remnants in the cavity ring (arrow) beside the area of high meristematic activity (dashed circle) and against the host bark (Hb). (E) Longitudinal section through the seedling showing initial penetration of the host bark (Hb) by the parasite endophyte (e), which has not yet reached the cambium zone (***), nor the host xylem (Hx). Notice also the elevation of the host bark (arrows) into the former cavity ring of the holdfast. (F) MicroCT scan of a Lugol-treated sample showing high density of holdfast (ho) and hypocotyl (hy) tissues indicating the presence of starch. Young endophyte (e) not yet in contact with the host cambium (***) or xylem (Hx). Low density in the region corresponding to the lines of crushed cells (black stars) indicating absence of starch. Black scale bars=1 mm; white scale bar=100 μm.

Selected seedling samples observed to reach the end of the second developmental stage (contact with the host surface and/or early holdfast formation) underwent morphological analyses. MicroCT scanning revealed that the holdfast had a concave shape, with its edges physically in contact with the host bark, and the centre of the structure forming a cavity (Fig. 3A). At the dome of the holdfast and across the hypocotyl, multiple high-density spots were detected (Fig. 3A, B). Through anatomical cross-sections, these structures were identified as vessel elements undergoing profuse differentiation (Fig. 3C). Noticeably, vessel differentiation was observed to stop at the upper region of the holdfast, where rows of crushed cells became clearly visible. Flanked by these two rows, a region of intense meristematic activity was observed at the centre of the holdfast, which then came into physical contact with the host bark as it prepared for penetration. With the formation of this penetration peg, the former cavity space in the holdfast became a cavity ring between the inner and outer parts of the holdfast (Fig. 3D). At this stage, the underlying area of the host branch exhibited slight irregularities in the bark, indicating some detachment of the still present host epidermis. The formation of the cavity ring, as a sign of full contact between the holdfast and the host branch, marks the transition from the second to the third developmental stage.

The holdfast continued to grow after being initially established. As a result, the volume of the structure markedly increased. Towards the end of the third stage, parasite tissue began penetrating through the host bark (Fig. 3E). In samples treated with Lugol’s solution prior to microCT scanning, the mistletoe penetration peg appeared as a high-density tissue, which indicates an abundance of starch granules (Supporting Fig. S3 at Zenodo). Both the hypocotyl and the holdfast also had observable starch, except in the region where lines of crushed cells were observed (Fig. 3F). Around the penetration site, the host bark bulged outwards, resulting in slight elevation underneath the penetration site. The remaining areas of the holdfast were in direct contact with the host cortex and, by interlocking with the host bark, eliminated the cavities at the interface.

**Fig. 4.**
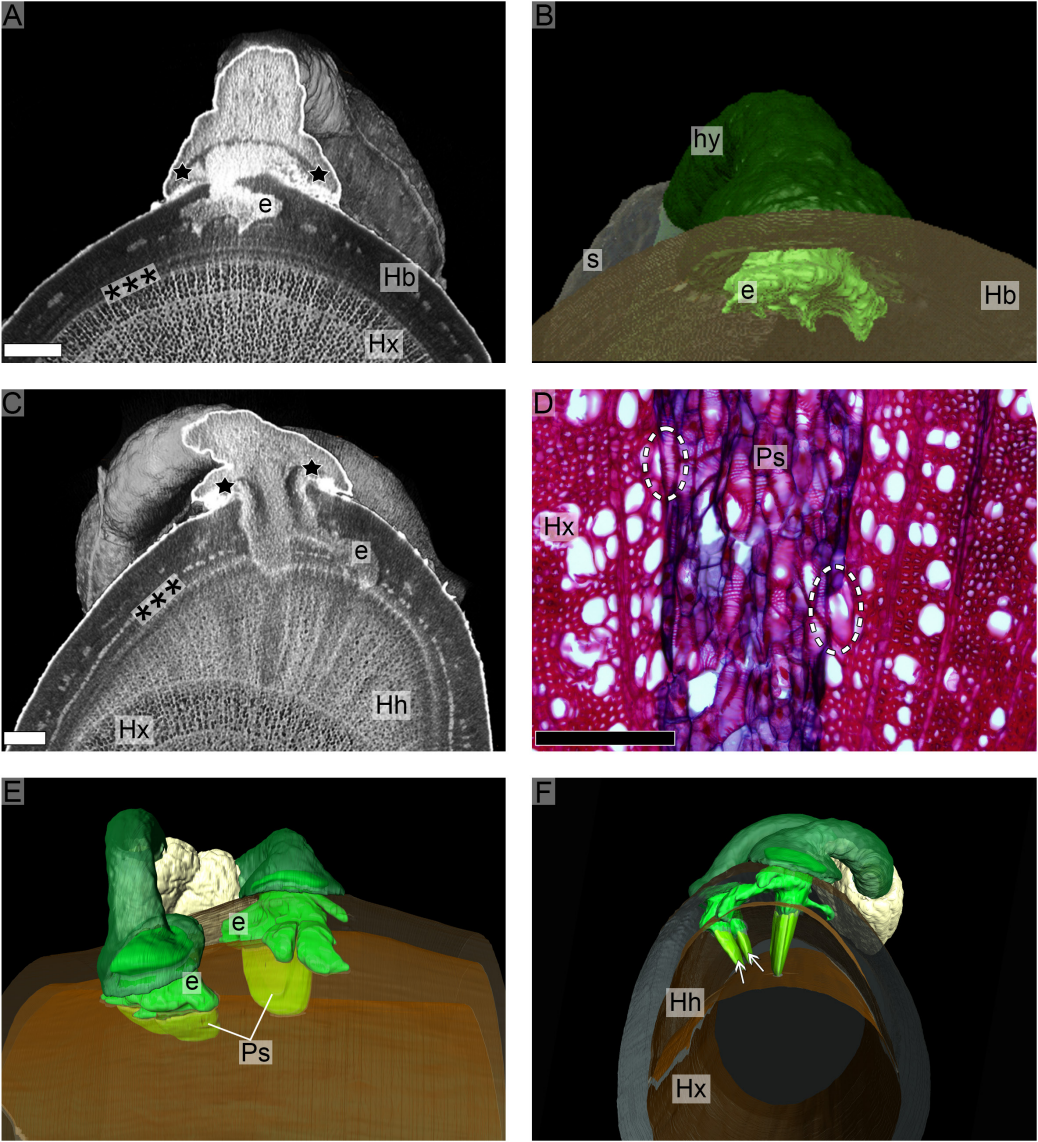
Morphology of the seedling at later developmental stages. (A) MicroCT scan showing expansion of the endophyte (e) within the host bark (Hb) prior to contact with host cambium zone (***) and xylem (Hx). Notice lines of crushed cells still visible (stars). (B) Virtual segmentation showing the three-dimensional expansion of the endophyte (e). Notice bent hypocotyl (hy) and the seed (s) still attached to host bark (Hb). (C) MicroCT showing initial contact between parasite endophyte (e) and host cambium zone (***). Lines of crushed cells (stars) become longer due to profuse endophyte proliferation. Notice host hypertrophy (Hh) in the most recent region of the host xylem (Hx). (D) Parasite sinker (Ps) embedded within the host xylem (Hx) forming vessel-to-vessel contact (dashed ellipses). (E) Virtual segmentation of a seed with two developing embryos whose endophyte (e) and sinkers (Ps) are not interconnected. (F) Virtual segmentation showing two sinkers (arrows) from the same seedling tissue within the hypertrophied (Hh) most recent annual ring on the host xylem (Hx). Black scale bar=200 μm; white scale bars=1 mm.

During the subsequent development, the host tissues below the penetration site elevated even further, making the holdfast appear flatter against the host surface. At the same time, the penetration peg continued to grow into the host bark, developing a complex endophyte tissue that also spread laterally in different directions (Fig. 4A). From the expanding area of the endophyte, further small, tapered protrusions continued to develop and reached the host cambium (Fig. 4B; Supporting Video SV1 at Zenodo). This contact with the host cambium marks the end of the third developmental stage. At the end of the 24 week study period, most of the samples were observed to have progressed to a fourth developmental stage, in which part of the endophyte tissue became embedded within the most recent annual ring formed by the host xylem (Fig. 4C). A clear difference in tissue density was observable between the surrounding host wood and the parasite tissue (Fig. 4C). It is through this part of the parasite tissue, which is known as a sinker, that a vessel-to-vessel connection with the host is established (Fig. 4D). In seedlings with more than one embryo, the endophytic system of each young plant develops with no detectable interconnections (Fig. 4E). On the other hand, a single young plant was observed to occasionally form two initial sinkers simultaneously (Fig. 4F). Hypertrophy of the host xylem was also clearly visible, altering the overall shape of the host branch (Fig. 4F). A comprehensive visualization of these geometrically complex structures can be found in Supporting Video SV2 at Zenodo.

### Biomechanics

For the first batch of seedlings (harvested in June–August 2023), 36 successful tensile tests were carried out on the seed, 13 successful tests were carried out on the hypocotyl, and 23 successful tests were carried out on the holdfasts. In the second batch (harvested in November 2023), only four successful hypocotyl tests and one successful holdfast test were performed. Fewer samples were tested in this batch because the seedlings had died and more samples were unsuitable for the tensile tests. In addition, the glue used in the holdfast tests limited the number of suitable samples, as more samples failed at the adhesive–holdfast interface rather than at the mistletoe–host interface, and could thus not be further evaluated.

**Fig. 5.**
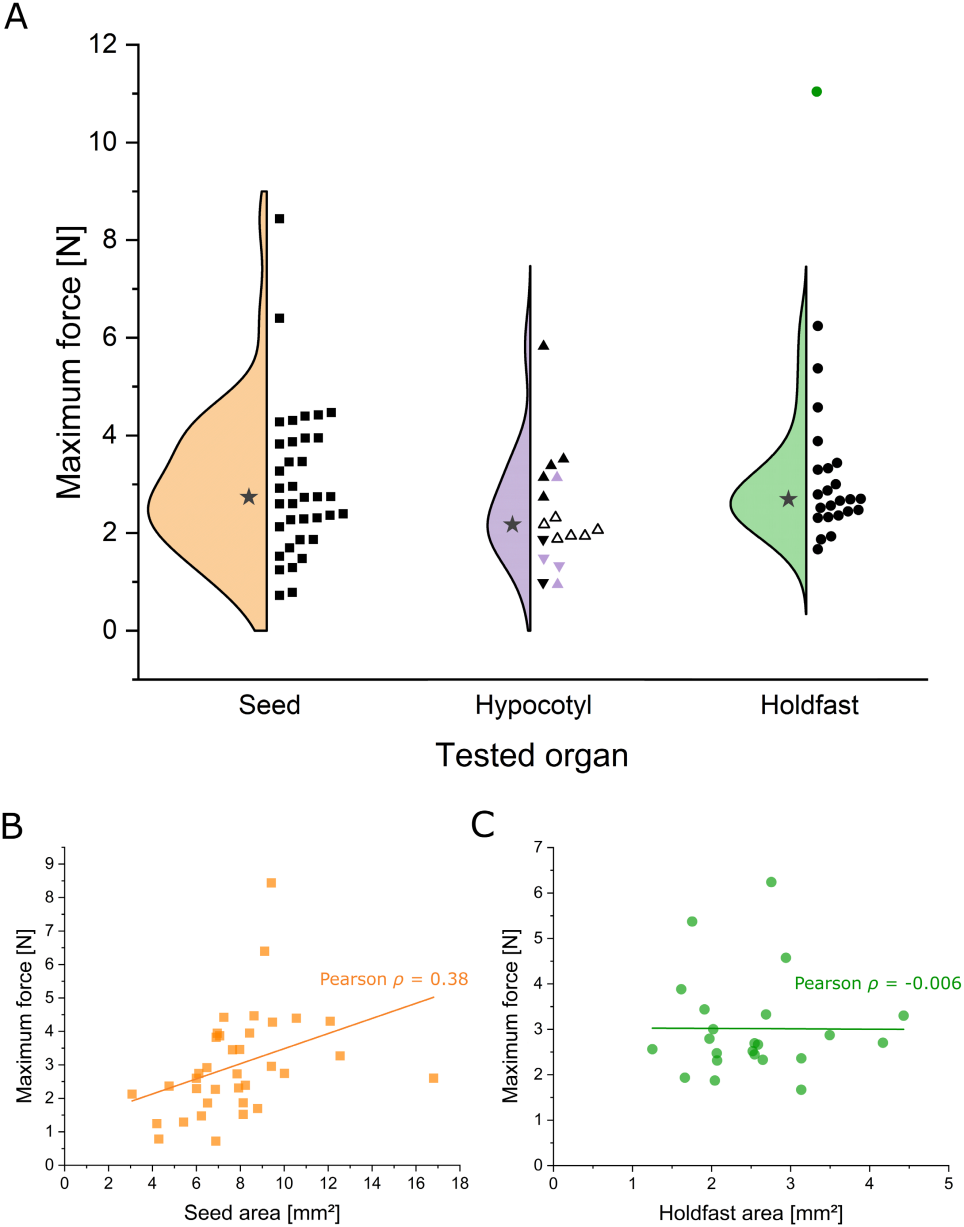
Maximum force data of biomechanical analysis of the three tested mistletoe organs. (A) Violin plot with individual data points of maximum forces from tensile tests on mistletoe seedling samples, divided according to the organ under load. The data points of the first batch are indicated by black symbols, and the data points of the second batch by coloured symbols. The left side of each group shows the data distribution of the first batch, with the median value marked as an asterisk. As the individual failure groups of the first batch of hypocotyl samples differ significantly from each other, they are labelled with different symbols (but were pooled for the presentation of the violin plot): white triangles indicate data points with failure at the holdfast, upright triangles indicate data points with failure at the hypocotyl, and inverted triangles indicate data points with failure at the seed. (B, C) Correlation analysis of the maximum force values of the tensile tests for the first batch of mistletoe seed samples (B) and the holdfast samples (C), with their corresponding contact area. The respective correlation coefficient of a linear regression is indicated.

During seed tests (*n*=36), ~14% of the samples detached completely from the host branch. In the remaining 86%, samples had partially detached, with part (~83%) or the entirety of the seed coat (~3%) still attached to the host branch. The maximum forces of these two failure groups (tissue rupture versus total rupture) were not significantly different (*P*=0.134, *U*-value=111, *Z*-value=1.510; combined median=2.74 N; Fig. 5A). The cross-sectional area of the seed attachment had a median value of 7.75 mm^2^ and varied between 3.07 mm^2^ and 16.80 mm^2^. Seed attachment area and maximum forces revealed a weak positive correlation (Pearson’s ρ=0.38, *F*-value: 5.57, Fig. 5B).

The hypocotyl experiments (*n*_total_=17) failed at three different sites. About 46% of the samples from the first batch and none of the samples from the second batch failed at the holdfast–host interface. Failures at the seed–host interface occurred in ~15% of the samples from the first batch and in 50% of the samples from the second batch. Finally, ~38% of the samples from the first batch and 50% of the samples from the second batch failed at the hypocotyl itself, with this structure being either pulled off the seed (in a total of six samples) or detached from the holdfast (in one sample). When comparing the maximum forces in the first batch of samples, we found a significant difference (*P*=0.004, *U*-value=0, *Z*-value= –2.347) between the samples that failed in the hypocotyl itself (median=3.38 N) and those that failed at the holdfast (median=2.00 N). No significant difference (*P*=0.095, *U*-value=10, *Z*-value=1.743) was found between the hypocotyl samples that failed in the hypocotyl itself and those that failed at the seed (median value of 1.37 N) and between those that failed at the holdfast and those that failed at the seed (*P*=0.333, *U*-value=4, *Z*-value=1.162). No statistical tests could be performed with data from the second batch due to its small sample size. However, a qualitative comparison of the data showed that the two batches did not differ markedly (see coloured versus black data points in Fig. 5A).

In the holdfast experiments (*n*_total_=24), ~87% of the samples from the first batch detached completely from the host branch. Among these, holdfast residues remained attached to the host branch after failure in ~13% of the cases. The one holdfast sample from the second batch also detached from the host, although no residues remained attached. No significant differences were found between the two failure types (partial detachment versus complete detachment) in the first batch of samples (*P*=0.698, *U*-value=25, *Z*-value= –0.411; combined median=2.69 N; Fig. 5A). In contrast to the statistical similarity between the two batches for the hypocotyl tests, the holdfast tested in the second batch had the highest maximum force value of all tested samples (11.1 N). It represents a clear outlier compared with all samples from the first batch and was therefore not included in the data for the comparison between groups. In fact, its value corresponds to 410.8% of the median value of the holdfast samples in the first batch, being 77.1% higher than their maximum value. Regarding holdfast cross-sectional area, values ranged between 1.25 mm^2^ and 4.43 mm^2^ with a median of 2.52 mm^2^. In the first batch, no correlation between holdfast attachment area and the maximum forces was detected (Pearson’s ρ= –0.006, *F*-value <0.001; Fig. 5C).

Finally, we compared the maximum force values among the three types of tests (seed×hypocotyl×holdfast). To obtain enough samples for statistical comparison, hypocotyl samples of both batches were pooled (despite the significant differences in their subgroups of the first batch; resulting in a pooled median value of 2.07 N) and compared with the seed and holdfast samples. No significant differences between the three groups were found (*P*=0.142, χ^2^=3.906; Fig. 3A). A detailed analysis of the strength values was not carried out because of measurement inaccuracies associated with normalizing the maximum forces across the functional contact areas, which were difficult to quantify. However, the strength values are used in the Discussion to provide a rough estimate of adhesion strength in comparison with other plants, such as climbing vines. A detailed overview of the raw data for each sample can be found in the Supporting Material at Zenodo.

## Discussion

Mistletoes are a widely diverse functional group of parasitic plants, brought together by their shared general life history and unique ability to develop entirely off the soil (Mathiasen *et al.*, 2008; Teixeira-Costa and Davis, 2021). This fully aerial lifestyle is strongly associated with the presence of the viscin tissue that coats the seed inside the fruit and facilitates initial attachment to the host surface (Kuijt, 2015). An upper estimate of the adhesive strength at the seed–host interface is provided by single-lap shear tests, in which two strips of a given material are joined, in this case by viscin from *V. album* fruits, and the shear forces required to separate them are measured. Compared with other materials, such as metal, glass, and plastic, viscin provided the best adhesion when in contact with wood, especially in a dry state (1.71 MPa), with adhesion decreasing gradually with increasing humidity (down to 0.34 MPa at 49% relative humidity) (Horbelt *et al.*, 2022; George *et al.*, 2024). This is made possible by a moisture-dependent reorientation of the cellulose fibrils, which indicates a degree of reversibility of viscin adhesion (Horbelt *et al.*, 2019).

These data are in good agreement with the values we measured, which resulted in a median strength (*F*_max_/*A*_contact_) of 0.37 MPa for the seed experiments. However, this value gives a lower estimate of the actual adhesion, as it takes into account the total contact area *A*_contact_ rather than the fact that the adhesion is actually provided by single viscin threads, which would result in lower *A*_contact_ values and thus higher strength. This also helps to explain the weak correlation between the contact area of the seed and the measured maximum forces, which would be expected to be higher if complete adhesion was present (Fig. 5B). Furthermore, the moisture dependence of mistletoe seed adhesion was qualitatively observed, as the seeds were found to be quite loosely attached to the host branch by just scanty viscin threads after rainfall but became firmly attached again after a few hours of drying. Considering that a period of months can elapse between seed dispersion and penetration of host tissues (Supporting Fig. S2 at Zenodo; Zuber, 2004), mistletoe establishment and survival depend strongly on viscin providing sufficient attachment strength for the seed during the coming developmental stages.

Nevertheless, viscin-mediated intermolecular adhesion might not be enough to secure the initial attachment of all mistletoe species. In several species of the genus *Psittacanthus* (Loranthaceae), for instance, a ‘latex-like’ substance is also exuded from the base of the seedling, further aiding in attachment to the host branch (Ornelas *et al.*, 2024a). Interestingly, many *Psittacanthus* species follow a pattern of seedling development in which no elongation of the hypocotyl is observed (Kuijt, 1970). In these plants, an often uneven viscin coating of the seeds within the fruit restricts attachment capacity to the basal portion of the seed (Kuijt, 2009; Ornelas *et al.*, 2024b). From this portion, the tip of the embryo emerges and expands to form a holdfast prior to haustorium penetration (Kuijt, 1970; Aguilar-Venegas *et al.*, 2023). The absence of an elongated hypocotyl is also reported for *Aetanthus*, the sister clade of *Psittacanthus*, and for the distantly related *Dendrophthoe* (Ang and Yong, 2005; Kuijt, 2014, 2015). In other Loranthaceae genera, hypocotyl size varies from short and robust, as in *Phthirusa* and *Struthanthus*, to long and slender, as in *Ligaria* and *Tripodanthus* (Kuijt, 1982). This variation can be at least partially due to the orientation of the mistletoe seed upon the host branch, which depends on the distribution of the viscin coating around the seed (Wilson and Calvin, 2006). Likewise, the abundance of viscin in the fruits of *Phoradendron* and *Viscum* species might explain the lack of variation regarding hypocotyl size and germination patterns among the Viscaceae (Kuijt, 2015). In members of this family, including *V. album*, the holdfast develops from the tip of an elongated hypocotyl. In this way, the hypocotyl forms a physiological and mechanical bridge between the first (seed surface) and second (holdfast) point of attachment established by the growing seedling of *V. album* with the surface of the host.

**Fig. 6.**
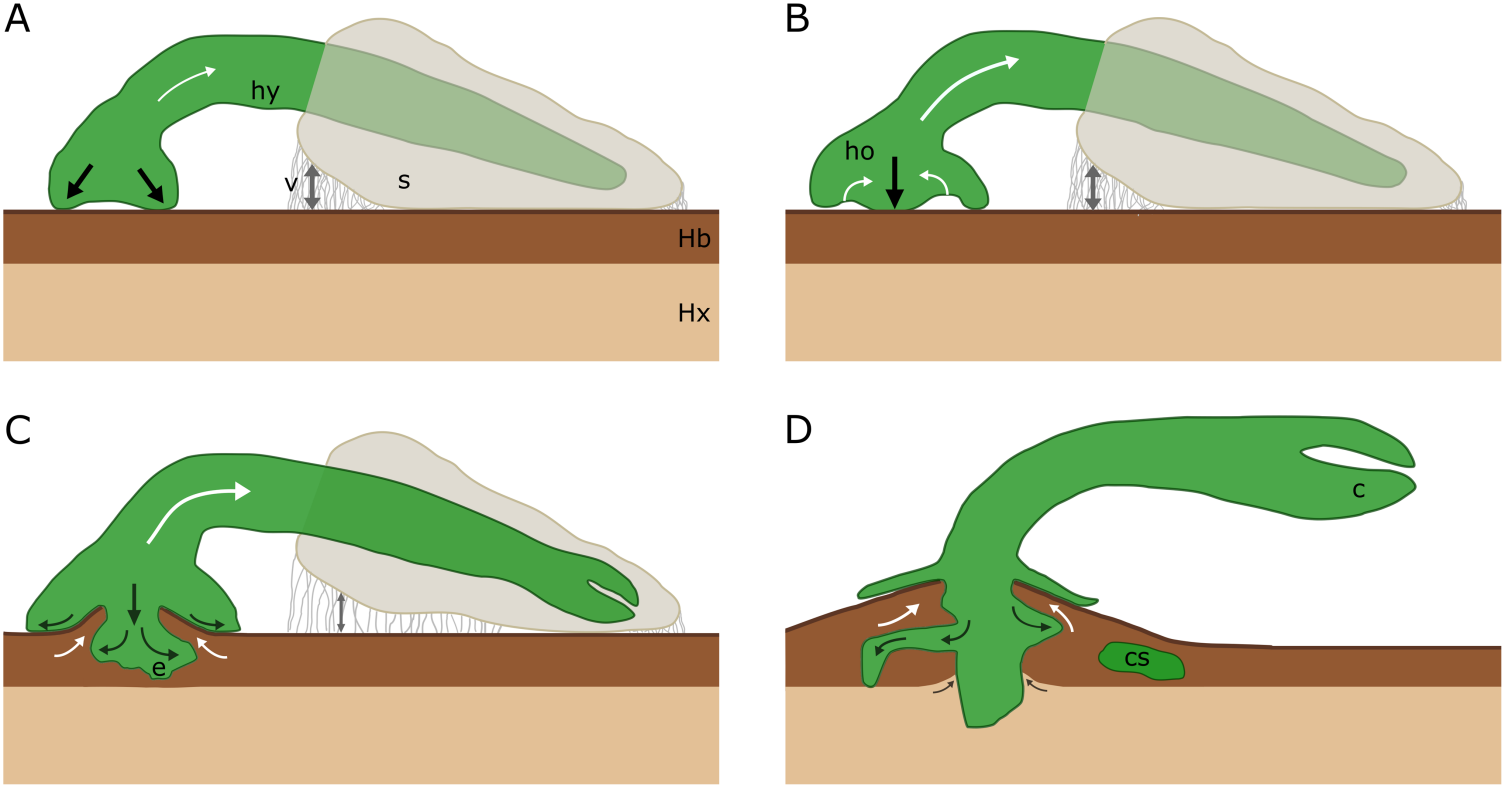
Schematic representation of the forces (arrows) acting during four stages of mistletoe establishment. (A) Upon contact, the edges of the hypocotyl (hy) tip exert pressure (growth-induced forces marked by black arrows) against the host bark (Hb), generating a reaction force (white arrows) in the opposite direction along the bridge-like structure of the hypocotyl. Intermolecular adhesive forces (grey arrows) caused by viscin threads keep the seed (s) in place and in contact with the host bark. (B) At a later stage, the penetration peg grows against the host bark, causing reaction forces in the opposite direction within the holdfast (ho), forming layers of collapsed cells. (C) The endophyte (e) proliferates and causes pressure within the host bark, which becomes dislocated towards the area previously corresponding to the holdfast cavity, creating an interlocking mechanism between mistletoe and host. Note that the attachment forces between the seed and the host bark diminish as fewer viscin threads are present. (D) In the following stages, besides the formation of cortical strands (cs) and the flattening of the holdfast, additional lateral growth of the endophyte can be observed, which further strengthens the interlocking mechanism between mistletoe and host. The secondary growth of the host branch causes pressure against the main sinker, resulting in slightly irregular growth along its xylem–bark interface.

In this context, we propose that the elongated hypocotyl facilitates a transition from attachment by viscin to attachment by mechanical penetration and further connection between mistletoe and host mediated by the holdfast and the endophyte. This interpretation is based on our mechanical analyses showing that the forces required to detach either the seed or the holdfast are almost identical during the initial developmental stages, with the hypocotyl being developed to a level where it can withstand similar forces, instead of acting as a weak point in the overall system (Fig. 5A). In this respect, the multistep attachment of the mistletoe seedling to the host differs from that of other climbing plants, in which the first steps are often characterized by lower adhesive forces (e.g. by a first formation of a weak attachment by microspines, as, for example, in *Mandevilla hirsuta*, or by weak chemical adhesion, as in the root/root hair system of English ivy or in the adhesive pads of the tendrils of some passion flowers), and full adhesion is only achieved in later stages (e.g. by twining or growing into the attachment substrate, increased chemical adhesion, and lignification of the attachment organs) (Melzer *et al*., 2010; Bohn *et al*. 2015; Klimm *et al*., 2022; Lehnebach *et al*., 2022). However, unlike the mistletoe seedling, a single unsuccessful attachment event is not catastrophic for them due to the redundancy provided by multiple attachment roots/root hairs, pads, attachment leaves, or attachment stems, as well as the physiological and mechanical anchoring already established in the soil. In contrast, the fact that mistletoe establishes a mechanical balance between the attachment structures from the earliest stage shows how well this system is adapted to avoid catastrophic behaviour and ensure a mechanically safe transition to a state of full physiological and structural connection with the host. The slightly lower maximum forces in the hypocotyl experiments can be explained by the fact that, unlike in the holdfast and seed tests, it was not possible to ensure that the tensile forces acted perfectly perpendicular to the adhesive structures due to the more complex geometry and tensile setup, resulting in additional shear forces (Fig. 1). Furthermore, the bridge-like structure formed by the hypocotyl might help to provide the required support for holdfast development (Thoday, 1951; Sallé, 1983). This is accomplished by counteracting the mechanical forces generated during haustorium penetration into the host tissue and transmitting them to the viscin-adhering seeds as a connecting lever arm (Fig. 6). This implies that viscin adhesion not only plays a role in the initial germination process, when the holdfast is not yet developed, but also plays a crucial role later in the transition to a holdfast-dominated attachment. In the absence of an elongated hypocotyl associated with the seed, seedlings of *Psittachanthus* would require additional adhesion at the penetration site.

Upon contact, the sides of the hypocotyl convex tip grow tangentially to flatten out the structure against the surface of the host branch, forming a dome-shaped holdfast (Thoday, 1951). Here, we show that the holdfast of *V. album* is not entirely flattened out against the host surface, instead assuming an initially concave shape (Fig. 6A). The cavity formed by this concave shape allows space for the growth and action of a penetrating body, which develops at the centre of the holdfast (Fig. 6B). At later developmental stages, the remaining space in this cavity allows an interlocking of the holdfast and the host bark (Fig. 6C), which further strengthens the mistletoe–host attachment. This is supported by the marked difference in the strength values of the holdfast between samples in the first and second batches. In the first batch, when samples had just begun penetrating into host tissue, the median force value was 1.12 MPa, compared with 2.65 MPa recorded for samples in the second batch, which were collected 3–4 months later.

At even later developmental stages, the mean strength values for mistletoe–host connections of *V. album* were found to be 1.24 MPa for entire samples and 1.61 MPa for cut slices (Mylo *et al.*, 2022), independently of the mistletoe age. The synthesis of data from holdfast experiments and those from developed mistletoe indicates that a mechanically robust connection is established between the mistletoe and its host at the earliest stages of the physiological connection. Despite the substantial growth of the mistletoe and its connection with the host (Mylo *et al.*, 2021), the strength values remain at a similar level for more than two decades. The adhesive strength of the holdfast is markedly higher than that of the adhesive pads of passion flower (*Passiflora discophora*, Passifloraceae) (0.35–0.66 MPa; Klimm *et al.*, 2022) and is within the range of the adhesive pads of Boston ivy (*Parthenocissus tricuspidata*, Vitaceae) (1.53 MPa; Steinbrecher *et al.*, 2010). In contrast to the macro- and microscopic physiological connection between mistletoe and host, these attachment structures of climbing lianas are mostly chemically bonded to their attachment substrates, with some physical interlocking at the subcellular level, resulting in a good firm closure. Their redundant arrangement of multiple attachment pads on the tendrils ensures that the failure of individual pads does not result in the complete failure and subsequent death of the plant (Mylo and Speck, 2023), as would be the case in the event of the mistletoe’s holdfast breaking off. It is noteworthy that, although *V. album* is reported to produce adventitious stems from the endophyte tissue (Tubeuf, 1923; and references since then), this is documented to occur as a response to the dieback of an already established main stem (Wangerin, 1937; Wallden, 1961). Therefore, securing the establishment of the seedling-borne stem is paramount for the survival of *V. album*.

The marked increase in the tensile forces required for the holdfast structure to fail when comparing summer-harvested (first batch) and winter-harvested (second batch) samples (Fig. 5A) suggests that, following an initial equilibrium between the tissues involved, the holdfast develops as the dominant attachment structure after a few months and forms an increasingly stronger connection with the host through additional growth. The significance of seed adhesion subsequently diminishes over the winter, resulting in seed detachment from the host branch and release of the cotyledons from the seed coat (Fig. 6D). In the following spring, seedling growth is characterized by intense endophyte proliferation within the host bark and the formation of an intercalary meristem at the interface with the host cambium (Sallé, 1978, 1979). This coincides with the period of cambial reactivation in deciduous angiosperms grown in seasonally cold climates (Savage and Chuine, 2021), which correspond to the majority of host species reported for *V. album* subsp. *album* (Barney *et al.*, 1998).

## Conclusion

Here, we have shown that the maximum forces created by the seedling of *V. album* exceed the weight of the plant by a multiple, as young seedlings weigh much less than 1 g, which is equivalent to 0.01 N. This can be explained by the fact that the plant has to deal with additional forces in addition to its own weight, for example from the wind and, above all, from the counterforce when penetrating the host. Considering the forces of ~2 N that are necessary to cause failure in the three attachment tissues, these structures are capable of supporting a mass of 200 g under static loads. With a weight of <1 g, a safety factor of >200 for young mistletoe seedlings is guaranteed. This allows for the full functionality of the seedlings to be guaranteed even under more dynamic loads, such as gusts of wind or climbing animals and birds. Furthermore, attachment structures in nature have to withstand not only tensile forces but also shear and compression forces.

The absence of a correlation between the maximum forces and the size of the holdfast (Fig. 5C) demonstrates the intricate processes that occur during the described developmental stages. The individual stages in this process are distinguished by varying degrees of cavity formation, chemical bonding, and cellular connections, and thus the absolute size of the holdfast in the young seedling stages is not indicative of its adhesive force. Considering the importance of the holdfast for seedling development in *V. album*, we believe that further research into the biomechanics of seedling development in other mistletoe species with different hypocotyl sizes, such as those grouped in the genera *Psittachanthus*, *Struthanthus*, *and Tripodanthus*, would provide a better understanding of the relevance of holdfast elongation in mistletoe evolution. This is especially interesting considering some of the significant changes proposed in more recent phylogeny treatments (Liu *et al.*, 2018; Nickrent *et al.*, 2019) compared with the work of Wilson and Calvin (2006). Moreover, given that viscin composition differs between Viscaceae and Loranthaceae mistletoes (Gedalovich *et al.*, 1988; Gedalovich-Shedletzky *et al.*, 1989; Lichter and Berry, 1991) and that its adhesion strength varies between dry and wet conditions (Horbelt *et al.*, 2022; George *et al.*, 2024), research into the attachment properties of this tissue in *Psittacanthus* species inhabiting areas with different climates could provide greater insight into the potential applications of this biological material.

## Data Availability

All data supporting the findings of this study are available within the paper and within its supplementary materials published in Zenodo (https://zenodo.org/records/14179266).

## References

[CIT0001] Aguilar-Venegas M , Quintana-Rodríguez E, Aguilar-Hernández V, López-García CM, Conejo-Dávila E, Brito-Argáez L, Loyola-Vargas VM, Vega-Arreguín J, Orona-Tamayo D. 2023. Protein profiling of *Psittacanthus calyculatus* during mesquite infection. Plants 12, 464. 36771550 10.3390/plants12030464 PMC9920738

[CIT0002] Albert M , Belastegui-Macadam X, Kaldenhoff R. 2006. An attack of the plant parasite *Cuscuta reflexa* induces the expression of attAGP, an attachment protein of the host tomato. The Plant Journal 48, 548–556. 17076801 10.1111/j.1365-313X.2006.02897.x

[CIT0003] Ang SLP , Yong JWH. 2005. A protocol for in vitro germination and sustainable growth of two tropical mistletoes. Plant Cell, Tissue and Organ Culture 80, 221–228.

[CIT0004] Aukema JE. 2003. Vectors, viscin, and Viscaceae: mistletoes as parasites, mutualists, and resources. Frontiers in Ecology and the Environment 1, 212–219.

[CIT0005] Barney CW , Hawksworth FG, Geils BW. 1998. Hosts of *Viscum album*. European Journal of Forest Pathology 28, 187–208.

[CIT0006] Bohn HF , Günther F, Fink S, Speck T. 2015. A passionate free climber: structural development and functional morphology of the adhesive tendrils in *Passiflora discophora*. International Journal of Plant Sciences 176, 294–305.

[CIT0007] Furuhashi T , Furuhashi K, Weckwerth W. 2011. The parasitic mechanism of the holostemparasitic plant *Cuscuta*. Journal of Plant Interactions 6, 207–219.

[CIT0008] Gedalovich E , Kuijt J. 1987. An ultrastructural study of the viscin tissue of *Phthirusa pyrifolia* (H.B.K.) Eichler (Loranthaceae). Protoplasma 137, 145–155.

[CIT0009] Gedalovich E , Kuijt J, Carpita NC. 1988. Chemical composition of viscin, an adhesive involved in dispersal of the parasite *Phoradendron californicum* (Viscaceae). Physiological and Molecular Plant Pathology 32, 61–76.

[CIT0010] Gedalovich-Shedletzky E , Delmer DP, Kuijt J. 1989. Chemical composition of viscin mucilage from three mistletoe species—a comparison. Annals of Botany 64, 249–252.

[CIT0011] George SD , Andraos E, Priemel T, Horbelt N, Keiser G, Kumar A, Heiss C, Gierlinger N, Azadi P, Harrington MJ. 2024. Structure, function, and application of self-healing adhesives from mistletoe viscin. Advanced Functional Materials 34, 2307955.

[CIT0012] Gorb SN. 2008. Biological attachment devices: exploring nature’s diversity for biomimetics. Philosophical Transactions of the Royal Society A: Mathematical, Physical, and Engineering Sciences 366, 1557–1574. 10.1098/rsta.2007.2172 18192171

[CIT0013] Groot EP , Sweeney EJ, Rost TL. 2003. Development of the adhesive pad on climbing fig (*Ficus pumila*) stems from clusters of adventitious roots. Plant and Soil 248, 85–96.

[CIT0014] Horbelt N , Eder M, Bertinetti L, Fratzl P, Harrington MJ. 2019. Unravelling the rapid assembly process of stiff cellulosic fibers from mistletoe berries. Biomacromolecules 20, 3094–3103. 31314500 10.1021/acs.biomac.9b00648

[CIT0015] Horbelt N , Fratzl P, Harrington MJ. 2022. Mistletoe viscin: a hygro- and mechano-responsive cellulose-based adhesive for diverse material applications. PNAS Nexus 1, pgac026. 36712808 10.1093/pnasnexus/pgac026 PMC9802232

[CIT0016] Kampowski T , Mylo MD, Speck T, Poppinga S. 2017. On the morphometry, anatomy and water stress behaviour of the anisocotyledonous *Monophyllaea horsfieldii* (Gesneriaceae) and their eco-evolutionary significance. Botanical Journal of the Linnean Society 185, 425–442.

[CIT0017] Klimm F , Schmier S, Bohn HF, Kleiser S, Thielen M, Speck T. 2022. Biomechanics of tendrils and adhesive pads of the climbing passion flower *Passiflora discophora*. Journal of Experimental Botany 73, 1190–1203. 34673926 10.1093/jxb/erab456 PMC8866636

[CIT0018] Klimm F , Thielen M, Homburger J, Modert M, Speck T. 2024. Natural coil springs: biomechanics and morphology of the coiled tendrils of the climbing passion flower *Passiflora discophora*. Acta Biomaterialia 189, 478–490. 39393657 10.1016/j.actbio.2024.10.002

[CIT0019] Ko SM , Kwon YK, Kim JH, Song IJ, Lee HY, Choi DW, Liu JR, Kim SW. 2014. Transcriptome analysis of mistletoe (*Viscum album*) haustorium development. Horticulture, Environment and Biotechnology 55, 352–361.

[CIT0020] Kuijt J. 1969. The biology of parasitic flowering plants. Berkeley, CA: University of California Press.

[CIT0021] Kuijt J. 1970. Seedling establishment in *Psittacanthus* (Loranthaceae). Canadian Journal of Botany 48, 705–711.

[CIT0022] Kuijt J. 1982. Seedling morphology and its systematic significance in Loranthaceae of the New World, with supplementary comments on Eremolepidaceae. Botanische Jahrbucher 103, 305–342.

[CIT0023] Kuijt J. 2009. Monograph of *Psittacanthus* (Loranthaceae). Systematic Botany Monographs 86, 1–361.

[CIT0024] Kuijt J. 2014. A monograph of the genus *Aetanthus* (Loranthaceae). Plant Diversity and Evolution 131, 1–51.

[CIT0025] Kuijt J. 2015. Santalales. In: Kubitzki K, ed. The families and genera of vascular plants. Volume XII flowering plants. Eudicots. Santalales, Balanophorales. Cham: Springer International Publishing, 209.

[CIT0026] Lamont B. 1983. Germination of mistletoes. In: Calder DM, Bernhardt P, eds. The biology of mistletoes. Sydney: Academic Press.

[CIT0027] Lehnebach R , Paul-Victor C, Courric E, Rowe NP. 2022. Microspines in tropical climbing plants: a small-scale fix for life in an obstacle course. Journal of Experimental Botany 73, 5650–5670. 35562069 10.1093/jxb/erac205 PMC9467647

[CIT0028] Lichter JM , Berry AM. 1991. Establishment of the mistletoe *Phoradendron macrophyllum*: phenology of early stages and host compatibility studies. Botanical Gazette 152, 468–475.

[CIT0029] Liu B , Le CT, Barrett RL, Nickrent DL, Chen Z, Lu L, Vidal-Russell R. 2018. Historical biogeography of Loranthaceae (Santalales): diversification agrees with emergence of tropical forests and radiation of songbirds. Molecular Phylogenetics and Evolution 124, 199–212. 29550535 10.1016/j.ympev.2018.03.010

[CIT0030] Mathiasen RL , Shaw DC, Nickrent DL, Watson DM. 2008. Mistletoes: pathology, systematics, ecology, and management. Plant Disease 92, 988–1006. 30769529 10.1094/PDIS-92-7-0988

[CIT0031] Melzer B , Steinbrecher T, Seidel R, Kraft O, Schwaiger R, Speck T. 2010. The attachment strategy of English ivy: a complex mechanism acting on several hierarchical levels. Journal of the Royal Society Interface 7, 1383–1389. 20462880 10.1098/rsif.2010.0140 PMC2894893

[CIT0032] Mylo MD , Hofmann M, Balle F, Beisel S, Speck T, Speck O. 2022. Biomechanics of the parasite–host interaction of the European mistletoe. Journal of Experimental Botany 73, 1204–1221. 34849736 10.1093/jxb/erab518 PMC8866656

[CIT0033] Mylo MD , Hofmann M, Delp A, Scholz R, Walther F, Speck T, Speck O. 2021. Advances on the visualization of the internal structures of the European mistletoe: 3D reconstruction using microtomography. Frontiers in Plant Science 12, 715711. 34616413 10.3389/fpls.2021.715711 PMC8488221

[CIT0034] Mylo MD , Speck O. 2023. Longevity of system functions in biology and biomimetics: a matter of robustness and resilience. Biomimetics 8, 173. 37092425 10.3390/biomimetics8020173 PMC10123643

[CIT0035] Nickrent DL , Anderson FE, Kuijt J. 2019. Inflorescence evolution in Santalales: integrating morphological characters and molecular phylogenetics. American Journal of Botany 106, 402–414. 30856677 10.1002/ajb2.1250

[CIT0036] Olsen S , Krause K. 2017. Activity of xyloglucan endotransglucosylases/hydrolases suggests a role during host invasion by the parasitic plant *Cuscuta reflexa*. PLoS One 12, e0176754. 28448560 10.1371/journal.pone.0176754 PMC5407826

[CIT0037] Ornelas JF , Galicia S, Vásquez-Aguilar AA, Vovides AP. 2024a. Fruit anatomy and seedlings of the mistletoe *Psittacanthus schiedeanus* (Loranthaceae). Botany 102, 147–159.

[CIT0038] Ornelas JF , Lara C, Morales-Saldaña S, et al 2024b. Insights into mistletoe seed germination: a study of hemiparasitic *Psittacanthus* Mart. (Santalales: Loranthaceae) mistletoes. Flora 316, 152527.

[CIT0039] Rowe NP , Isnard S, Speck T. 2004. Diversity of mechanical architectures in climbing plants: an evolutionary perspective. Journal of Plant Growth Regulation 23, 108–128.

[CIT0040] Rowe NP , Speck T. 2015. Stem biomechanics, strength of attachment, and developmental plasticity of vines and lianas. In: Schnitzer S, Bongers F, Burnham R, Putz F eds. The ecology of lianas. Chichester, UK: Wiley-Blackwell, 323–341.

[CIT0041] Ruzin SE. 1999. Plant microtechnique and microscopy. Oxford, UK: Oxford University Press.

[CIT0042] Sallé G. 1978. Origin and early growth of the sinkers of *Viscum album* L. Protoplasma 96, 267–273.

[CIT0043] Sallé G. 1979. Le systeme endophytique du *Viscum album*: anatomie et fonctionnement des suçoirs secondaires. Canadian Journal of Botany 57, 435–449.

[CIT0044] Sallé G. 1983. Germination and establishment of *Viscum album* L. In: Calder MC, Bernhardt P, eds. The biology of mistletoes. Sydney: Academic Press, 145–159.

[CIT0045] Savage JA , Chuine I. 2021. Coordination of spring vascular and organ phenology in deciduous angiosperms growing in seasonally cold climates. New Phytologist 230, 1700–1715. 33608961 10.1111/nph.17289

[CIT0046] Seidelmann K , Melzer B, Speck AT. 2012. The complex leaves of the Monkey’s comb (*Amphilophium crucigerum*, Bignoniaceae): a climbing strategy without glue. American Journal of Botany 99, 1737–1744. 23092993 10.3732/ajb.1200288

[CIT0047] Spicer ME , Ortega J. 2023. Source height and contact with terrestrial soil drive transplanted epiphyte performance. Journal of Ecology 111, 2388–2400.

[CIT0048] Steinbrecher T , Danninger E, Harder D, Speck T, Kraft O, Schwaiger R. 2010. Quantifying the attachment strength of climbing plants: a new approach. Acta Biomaterialia 6, 1497–1504. 19818882 10.1016/j.actbio.2009.10.003

[CIT0049] Tada Y , Sugai M, Furuhashi K. 1996. Haustoria of *Cuscuta japonica*, a holoparasitic flowering plant, are induced by the cooperative effects of far-red light and tactile stimuli. Plant and Cell Physiology 37, 1049–1053.

[CIT0050] Tay JYL , Kovalev A, Zotz G, Einzmann HJR, Gorb SN. 2022. Holding on or falling off: the attachment mechanism of epiphytic *Anthurium obtusum* changes with substrate roughness. American Journal of Botany 109, 874–886. 35608083 10.1002/ajb2.16000

[CIT0051] Tay JYL , Zotz G, Einzmann HJR. 2023. Smoothing out the misconceptions of the role of bark roughness in vascular epiphyte attachment. New Phytologist 238, 983–994. 36775857 10.1111/nph.18811

[CIT0052] Teixeira-Costa L. 2021. A living bridge between two enemies: haustorium structure and evolution across parasitic flowering plants. Revista Brasileira de Botanica 44, 165–178.

[CIT0053] Teixeira-Costa L. 2022. Leveraging micro-CT scanning to analyze parasitic plant–host interactions. Journal of Visualized Experiments 179, e63423. 10.3791/63423 PMC9290312 35098949

[CIT0054] Teixeira-Costa L , Davis CC. 2021. Life history, diversity, and distribution in parasitic flowering plants. Plant Physiology 187, 32–51. 35237798 10.1093/plphys/kiab279 PMC8418411

[CIT0055] Thoday D. 1951. The haustorial system of *Viscum album*. Journal of Experimental Botany 2, 1–19.

[CIT0056] Tubeuf KF. 1923. Monographie der Mistel. München: R. Oldenbourg.

[CIT0057] Vaughn KC. 2002. Attachment of the parasitic weed dodder to the host. Protoplasma 219, 227–237. 12099223 10.1007/s007090200024

[CIT0058] Wallden B. 1961. Misteln vid dess nordgräns. Svensk Botanisk Tidskrift 55, 427–549.

[CIT0059] Wang A , Bose AK, Lehmann MM, Rigling A, Gessler A, Yu L, Li M. 2023. Water status and macronutrient concentrations, but not carbon status, of *Viscum album* ssp. *album* are determined by its hosts: a study across nine mistletoe–host pairs in central Switzerland. Frontiers in Plant Science 14, 1142760. 37223783 10.3389/fpls.2023.1142760 PMC10200922

[CIT0060] Wangering W. 1937. Loranthaceae. In: Kirchner OV, Loew E, Schröter C, eds. Lebensgeschichte der Blütenpflanzen Mitteleuropas. Stuttgart: Sammlung deutscher botanischer Zeitschriften, 953–1146.

[CIT0061] Wilson CA , Calvin CL. 2006. Character divergences and convergences in canopy-dwelling Loranthaceae. Botanical Journal of the Linnean Society 150, 101–113.

[CIT0062] Zuber D. 2004. Biological flora of Central Europe: *Viscum album* L. Flora 199, 181–203.

